# Central adiposity indices and inflammatory markers mediate the association between life’s crucial 9 and periodontitis in US adults

**DOI:** 10.1186/s12944-025-02619-1

**Published:** 2025-06-03

**Authors:** Jia-Jie Guo, Qi-Qi Hang, Ting Xu, Wei-Xuan Liang, Jia-Kun Gao, Hong-Biao Ou, Fu-Zhen Jiang, Xi-Chen-Hui Qiu, Zu-Zhang Tian, Yu-zhong Zhang, Jing Zhang

**Affiliations:** 1 The Second School of Clinical Medicine, Guangzhou Medical University, Guangzhou, 511436 China; 2 The Second School of Clinical Medicine, Nanjing Medical University, Nanjing, 211166 China; 3 School of Nursing, Nanjing Medical University, Nanjing, China; 4 The First School of Clinical Medicine, Guangzhou Medical University, Guangzhou, 511436 China; 5 Department of Artificial Intelligence, School of Automation, Hangzhou Dianzi University, Hangzhou, 310018 China; 6 Health Science Center, Shenzhen University, Shenzhen, China; 7 The Chinese University of Hong Kong, Shenzhen, China; 8 The Second Department of Infectious Disease, Shanghai Fifth People’s Hospital, Fudan University, 801 Heqing Road, Minhang District, Shanghai, 201100 China; 9 Center of Community-Based Health Research, Fudan University, 801 Heqing Road, Minhang District, Shanghai, 201100 China

**Keywords:** Periodontitis, Cardiovascular health, NHANES, Oral health, Abdominal fat, Central adiposity, Systemic inflammation response

## Abstract

**Background:**

Periodontitis, a chronic inflammatory disease, is closely linked to cardiovascular health. While Life’s Essential 8 (LE8) evaluates cardiovascular metrics, recent recommendations suggest incorporating psychological health (PHQ-9) to form Life’s Crucial 9 (LC9). However, evidence regarding the utility of LC9 in periodontal disease remains limited.

**Methods:**

We analyzed data from 7,674 adults in the 2009–2014 NHANES cycles. LC9 scores were calculated by integrating LE8 and PHQ-9 metrics, then categorized into quartiles. The association between LC9 and periodontitis was examined using weighted logistic regression, restricted cubic spline (RCS), subgroup and WQS analyses. Mediation analysis assessed the roles of central adiposity (ABSI, WWI) and systemic inflammation (SII, SIRI).

**Results:**

Higher LC9 scores were associated with lower periodontitis prevalence (29.4% in highest vs. 52.1% in lowest quartile; *P* < 0.001), with a 15.5% risk reduction per 10-unit increase (OR = 0.845; 95% CI: 0.795–0.897). WQS analysis identified nicotine exposure, sleep health, blood glucose, blood pressure, and depressive symptoms as key contributors. Mediation analysis showed partial effects through WWI (21.617%), ABSI (10.869%), SIRI (7.120%), and SII (5.351%). LC9 did not significantly outperform LE8 in prediction.

**Conclusions:**

Higher LC9 score is linked to reduced periodontitis prevalence and severity, with central adiposity and systemic inflammation partially mediating this relationship. These findings emphasize comprehensive cardiovascular health management may help reduce periodontal disease risk.

**Supplementary Information:**

The online version contains supplementary material available at 10.1186/s12944-025-02619-1.

## Background

Periodontitis, characterized by chronic inflammation leading to gradual degradation of periodontal tissues and alveolar bone, impacts over 40% of American adults. Growing evidence underscores its association with systemic disorders, notably cardiovascular diseases (CVD), mediated through overlapping inflammatory mechanisms [1, 2]. Contemporary diagnostic approaches combine conventional periodontal probing—probing pocket depth (PD) and clinical attachment level—with minimally invasive technologies such as ultrasonic imaging and Cone Beam Computed Tomography, enabling precise assessment while reducing patient discomfort [3]. In 2020, the American Heart Association (AHA) established Life’s Essential 8 (LE8) as a multidimensional framework for cardiovascular health (CVH) assessment, comprising eight modifiable domains: body mass index (BMI), physical activity, nicotine exposure, diet, sleep duration, blood cholesterol, blood glucose, and blood pressure. Recognizing the critical role of psychological well-being in CVH, the framework was recently expanded to Life’s Crucial 9 (LC9), which integrates mental health metrics with LE8 components [4]. Currently, this framework has not yet been formally incorporated into official guidelines. This advancement aligns with evidence that psychological distress may exacerbate periodontal disease through behavioral pathways (e.g., poor oral hygiene, smoking) and sustained hyperactivation of the hypothalamic-pituitary-adrenal (HPA) axis, thereby amplifying systemic inflammation [5, 6]. The Patient Health Questionnaire-9 (PHQ-9), a validated instrument for evaluating depression severity, enables systematic quantification of mental health status through its standardized assessment framework in epidemiological studies [7].

Recent studies have highlighted central adiposity as a strong predictor of periodontitis and CVD [8]. Recent developments in obesity metrics, including the a body shape index (ABSI) and weight-adjusted waist index (WWI), have facilitated expanded investigation into associations between adiposity and diverse pathological conditions [9]. In contrast to conventional anthropometric measures such as BMI that may not fully capture fat distribution patterns, or Waist-to-Height Ratio (WHtR) which primarily accounts for height proportionality without integrating total body weight, the WWI delivers a balanced evaluation of central obesity through the simultaneous incorporation of waist circumference (WC) and body weight parameters [10]. Meanwhile, ABSI quantifies the health risks associated with abdominal obesity by considering WC relative to height and BMI.

Emerging research underscores systemic inflammation as a potential mediator connecting periodontitis and metabolic disorders. Pro-inflammatory mediators including C-reactive protein (CRP), tumor necrosis factor-alpha (TNF-α), and interleukin-6 (IL-6) exhibit simultaneous upregulation in both periodontal and atherosclerotic tissues, suggesting overlapping inflammatory mechanisms between oral and systemic cardiovascular processes [11]. Novel biomarkers such as the systemic immune inflammation index (SII) and the systemic inflammation response index (SIRI) further corroborate this link, demonstrating significant associations with periodontitis severity [12]. As a primary contributor to low-grade systemic inflammation, central adiposity may serve as a critical mediator of the relationship between LC9 and periodontitis. Excess visceral fat promotes chronic inflammation via adipokine dysregulation and macrophage infiltration, exacerbating both periodontal and cardiovascular pathologies [13].

Despite these advances, critical gaps persist. First, the interplay between LC9-based CVH score and periodontitis remains underexplored, particularly regarding central adiposity and inflammation as a mediating mechanism. Second, while psychological health is now embedded in LC9, its role in modulating the periodontitis-CVH relationship—especially through PHQ-9-assessed depressive symptoms—has not been systematically evaluated.

The hypothesis suggests that central adiposity and systemic inflammation could mediate the link between LC9 score and periodontitis. Utilizing the NHANES data from 2009 to 2014, this study aims to: (1) Investigate the potential association between LC9 and periodontitis; (2) Evaluate the mediating effects of ABSI, WWI, SII, and SIRI on the LC9-periodontitis relationship; (3) Determine if the predictive efficacy of LC9 is superior to that of LE8. By elucidating these multidimensional interactions, our findings may advance integrative approaches to mitigate both oral and cardiovascular disease burdens through modifiable lifestyle and psychological interventions.

## Methods

### Study population

**Fig. 1:**
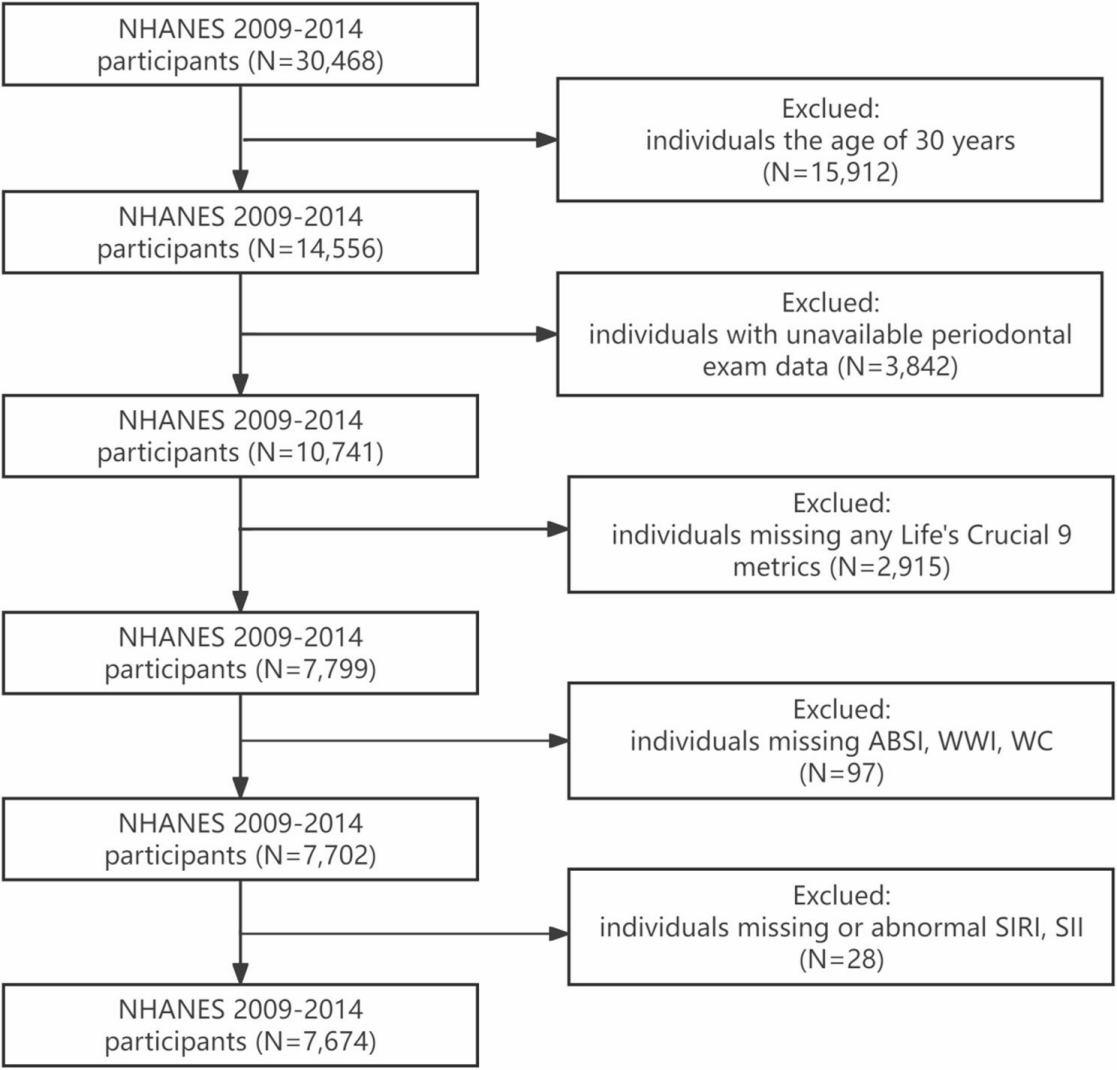
Flow chart of the sample selection

The NHANES is a nationally representative cross-sectional study conducted biennially to systematically assess health parameters and nutritional status within the civilian, non-institutionalized U.S. population [14]. The NHANES study protocol received ethical clearance from the National Center for Health Statistics (NCHS) Research Ethics Review Board (RERB), with all participants providing documented informed consent through written authorization processes [15]. The analytical cohort comprised 30,468 eligible individuals from the initial recruitment phase. Exclusion parameters consisted of the following: (1) individuals aged below 30 years; (2) those lacking accessible periodontal examination data; (3) participants with incomplete LC9 metrics; (4) individuals missing ABSl, WWI and WC; (5) individuals missing or abnormal SlRl and Sll. Following the application of these criteria, a final analytical sample of 7,674 participants was obtained, as depicted in Fig. 1.

### Periodontal examination and definitions

As in previous studies [16–18], periodontitis case definitions strictly followed Centers for Disease Control and Prevention (CDC) /American Academy of Periodontology (AAP) consensus criteria (Supplementary Material Text S1), with severity stratification (mild/moderate/severe) detailed in our methodological supplement. The primary exposure was dichotomized as periodontitis presence/absence per established epidemiological protocols [19], while mean PD and computed clinical attachment loss (CAL) served as continuous secondary outcomes.

### Life’s crucial 9 (LC9) score

The LC9 score, derived by averaging eight components of the LE8 and PHQ-9 score, encompasses nine distinct metrics: one psychological health metric, four health factors (blood glucose management, cholesterol control, blood pressure management, weight management), and four health behaviors (physical activity, healthy sleep, smoking cessation, healthy diet) [4, 7]. Detailed computational procedures for the LC9 metrics are provided in Supplementary Table S1. Individual components of the composite score are standardized on a 0-100 scale, with the final score calculated as the mean of individual component score. Dietary quality was evaluated using the HEI-2015, with specific scoring criteria and component details documented in Supplementary Table S2. Behavioral and psychological variables, including sleep patterns, smoking status, physical activity levels, and mental health indicators, were collected through validated questionnaires. Clinical measurements (blood glucose, blood pressure, BMI, and cholesterol) were obtained by certified personnel through NHANES protocols.

### Definition of mediating variables

The **WWI** was computed using the formula [20]:

$$\:\varvec{W}\varvec{W}\varvec{I}=\frac{\varvec{W}\varvec{C}}{\sqrt{\varvec{W}\varvec{e}\varvec{i}\varvec{g}\varvec{h}\varvec{t}}}$$

The **ABSI** was computed using the formula:

$$\:\varvec{A}\varvec{B}\varvec{S}\varvec{I}=\frac{\varvec{W}\varvec{C}}{{\varvec{B}\varvec{M}\varvec{I}}^{\frac{2}{3}}\:\times\:\sqrt{\varvec{H}\varvec{e}\varvec{i}\varvec{g}\varvec{h}\varvec{t}}}$$

incorporating both anthropometric and adiposity parameters [21].

The **SII** and **SIRI** were derived from peripheral blood cell counts as markers of immune-inflammatory status [22]. Their formulas are as follows:

$$\:\varvec{S}\varvec{I}\varvec{I}=\frac{\varvec{N}\varvec{e}\varvec{u}\varvec{t}\varvec{r}\varvec{o}\varvec{p}\varvec{h}\varvec{i}\varvec{l}\:\varvec{c}\varvec{o}\varvec{u}\varvec{n}\varvec{t}\times\:\varvec{P}\varvec{l}\varvec{a}\varvec{t}\varvec{e}\varvec{l}\varvec{e}\varvec{t}\:\varvec{c}\varvec{o}\varvec{u}\varvec{n}\varvec{t}\varvec{}}{\varvec{L}\varvec{y}\varvec{m}\varvec{p}\varvec{h}\varvec{o}\varvec{c}\varvec{y}\varvec{t}\varvec{e}\:\varvec{c}\varvec{o}\varvec{u}\varvec{n}\varvec{t}}$$

$$\:\varvec{S}\varvec{I}\varvec{R}\varvec{I}=\frac{\varvec{M}\varvec{o}\varvec{n}\varvec{o}\varvec{c}\varvec{y}\varvec{t}\varvec{e}\:\varvec{c}\varvec{o}\varvec{u}\varvec{n}\varvec{t}\times\:\varvec{N}\varvec{e}\varvec{u}\varvec{t}\varvec{r}\varvec{o}\varvec{p}\varvec{h}\varvec{i}\varvec{l}\:\varvec{c}\varvec{o}\varvec{u}\varvec{n}\varvec{t}\varvec{}}{\varvec{L}\varvec{y}\varvec{m}\varvec{p}\varvec{h}\varvec{o}\varvec{c}\varvec{y}\varvec{t}\varvec{e}\:\varvec{c}\varvec{o}\varvec{u}\varvec{n}\varvec{t}}$$

### Covariables

LC9 is a multifactorial score that itself incorporates multiple cardiovascular health behaviors and factors. Therefore, adding too many covariables to the analysis of LC9 score in relation to disease may complicate the model and obscure LC9’s own ability to comprehensively assess health risks [16, 17]. Drawing upon prior research and clinical experience [16, 17], the covariables considered encompass age, gender, race, family poverty-to-income ratio (PIR), marital status, educational attainment, alcohol use, health insurance status and use of dental floss/device. Details regarding these covariables are provided in Supplementary Text S2.

### Statistical analysis

Survey weights were incorporated for nationally representative estimates [23]. Given the findings of other researchers, we used WTMEC2YR/3 as the weighting factor to adjust the final sample size, incorporating the stratification variable SDMVSTRA, primary sampling unit SDMVPSU, and the two-year MEC examination weight WTMEC2YR for nationally representative estimates [18, 24–27]. To improve the robustness of the analysis and avoid bias associated with missing covariable information, missing covariable values were imputed and interpolated using the random forest algorithm [28, 29]. Baseline characteristics were summarized as continuous and categorical variables. Continuous variables were presented as mean and standard error (SE), with intergroup comparisons conducted through independent sample t-tests. Categorical measures were summarized as weighted proportions (absolute counts with percentages), with between-category differences assessed via Rao-Scott adjusted chi-square tests, incorporating complex survey design parameters. Associated probabilities (*P*-values) were computed for both analytical approaches to determine statistical significance. Four quartiles were established based on the distribution of LC9 score [7]. To evaluate the link between periodontitis and LC9, multivariable-adjusted logistic regression models were employed, wherein LC9 score were divided into quartiles. Trend consistency across quartiles was assessed using linear trend tests, with corresponding *P*-values quantifying the strength of association. Analytical models were implemented through a dual-phase approach: an unadjusted baseline model and a multivariable-adjusted model accounting for demographic (age, sex and race/ethnicity), socioeconomic (PIR, educational level and marital status), and behavioral/healthcare (alcohol consumption patterns, health insurance status and use of dental floss/device) covariables. To examine nonlinear associations between exposure and outcome variable, nonlinear associations were evaluated using RCS with four predefined knots. To identify potential inflection points indicative of threshold effects, segmented regression models were additionally employed. To explore heterogeneity across populations, subgroup analyses were conducted by age, gender, race, PIR, marital status, education level, alcohol consumption, health insurance status and use of dental floss/device [7]. To better understand the relative contribution of individual LC9 components to periodontitis risk, WQS regression was assessed [30]. Additionally, to examine associations between individual LC9 metrics and periodontitis, multivariable logistic regression was examined. ROC curve analysis assessed diagnostic efficacy disparities between LC9 and LE8 in periodontitis screening, with predictive superiority quantified through area under the curve (AUC) differentials.

In the sensitivity analyses, four complementary approaches were employed to verify the analytical consistency across methodological variations. First, to evaluate the association between LC9 and periodontitis severity, multinomial logistic regression was conducted, with periodontitis categorized into three levels: no periodontitis (reference group), non-severe periodontitis (mild or moderate), and severe periodontitis. Second, Second, associations between LC9 scores and continuous periodontal parameters, including mean PD and CAL, were examined using multivariable linear regression analyses. Third, to test the potential impact of imputation, we re-ran the multivariable logistic regression excluding participants with missing covariable data, without applying imputation techniques. Fourth, considering that the HEI-2015 score—a component of the LC9 metric—is derived from 24-hour dietary recall data, an additional sensitivity analysis was conducted using the corresponding dietary recall subsample weight (WTDRD1/3) in place of the examination weight (WTMEC2YR/3). The logistic regression model was repeated under this alternative weighting scheme, while keeping all covariates and model specifications consistent with the main analysis.

To clarify the mediating role of central adiposity indicators and systemic inflammation in the association between LC9 and periodontitis, a stepwise analytical approach was applied. First, to evaluate the eligibility of the proposed mediators—ABSI, WWI, SII and SIRI—weighted logistic regression models were used to examine their associations with periodontitis, adjusting for confounders. Subsequently, weighted linear regression analyses were conducted to assess the associations between LC9 and each of the four candidate mediators. Based on these preliminary results, suitable mediators were identified for the formal mediation analysis. Thereafter, path analysis was implemented under a counterfactual framework to quantify the direct, indirect, and total effects of LC9 on periodontitis through the selected mediators. The mediation proportion (MP), representing the relative contribution of the indirect effect, was calculated as the ratio of the indirect effect to the total effect using the formula:

$$\:MP=\left(\frac{Indirect\:effect}{Indirect\:effect+\:Direct\:effect}\right)\times\:100\%$$

All mediation analyses were performed using the R package *mediation* (v4.5.0), with 10,000 bootstrap iterations to generate bias-corrected confidence intervals (CIs) [27].

The EmpowerStats (www.empowerstats.com) software was utilized in conjunction with the R software (version 4.4.2) to process and analyze the entire dataset, with pre-specified significance criteria (α = 0.05, two-tailed).

## Results

### Study characteristics and age-adjusted prevalence of periodontitis

**Fig. 2:**
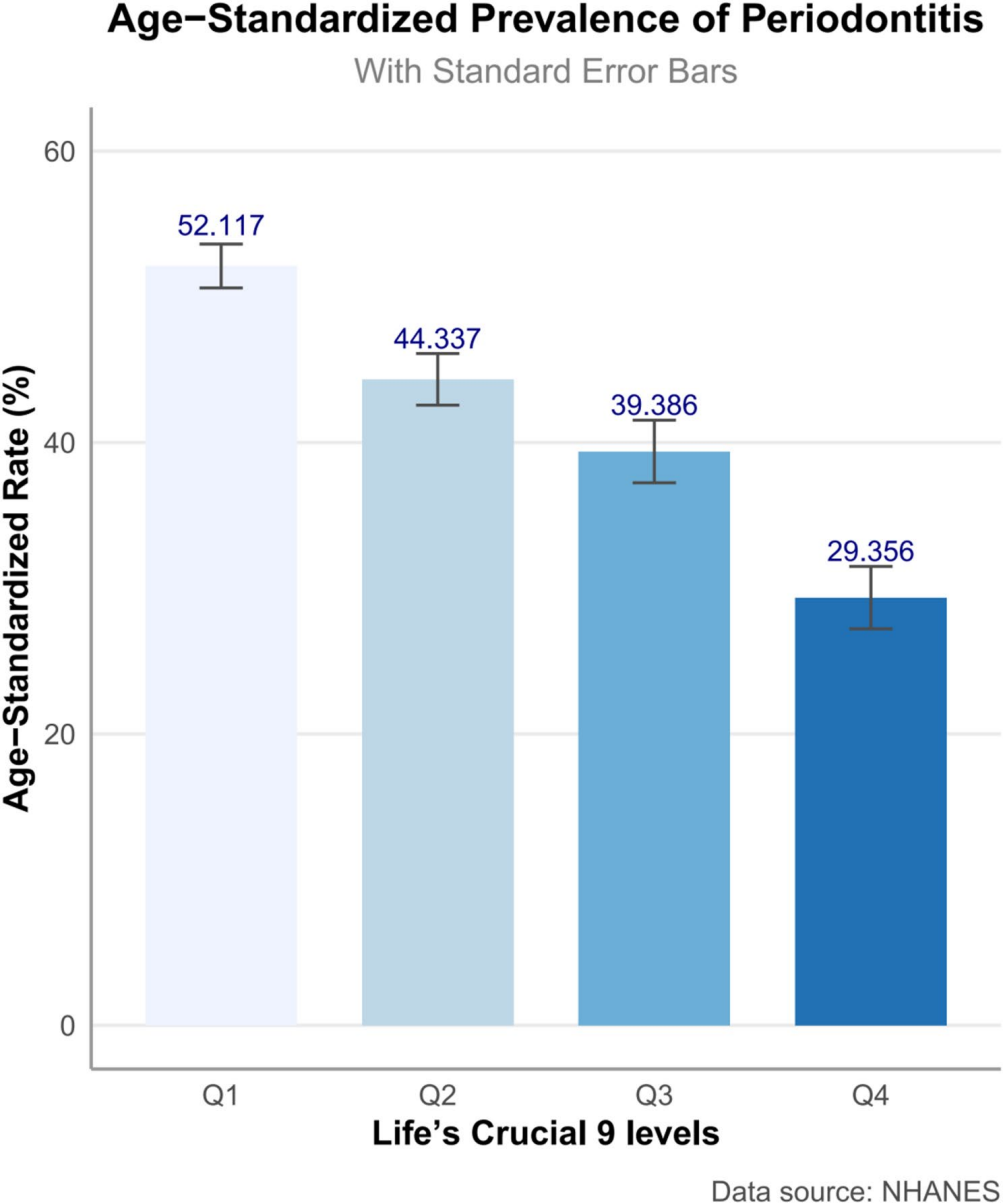
Age-adjusted prevalence of periodontitis in different levels of Life’s Crucial 9 score

The study cohort comprised 7,674 participants, averaging 51.260 (SE 0.249) years of age (Table 1). The cohort comprised 50.377% females, 71.886% non-Hispanic Whites, and 65.607% individuals with education beyond high school. The majority (70.806%) were married or cohabiting. Individuals with periodontitis demonstrated significant sociodemographic disparities relative to periodontitis-free counterparts, manifesting as advanced age, reduced educational attainment, and diminished household socioeconomic status. Participants without periodontitis displayed significantly elevated LC9 score across multiple domains, including diet, physical activity, BMI, lipid, and glucose metrics (all *P* < 0.05), whereas PHQ-9 score remained comparable between groups. Periodontitis participants had higher levels of central adiposity biomarkers WWI and ABSI. Periodontitis participants had higher levels of inflammatory biomarkers SIRI and SII. As shown in Fig. 2, the age-standardized prevalence of periodontitis demonstrated a clear inverse trend across quartiles of the LC9 score. Individuals in the lowest LC9 quartile (Q1) had the highest prevalence (52.117%), which progressively declined across Q2 (44.337%) and Q3 (39.386%), reaching the lowest prevalence in Q4 (29.356%).

**Table 1:** Baseline characteristics of the study population

Variables		Periodontitis		
	Overall	No	Yes	*P*-value^a^
**Age**,** years**	51.260 (0.249)	48.803 (0.322)	54.826 (0.379)	< 0.001
**PIR**	3.240 (0.052)	3.571 (0.055)	2.760 (0.057)	< 0.001
**WC**,** cm**	99.959 (0.282)	98.617 (0.317)	101.908 (0.372)	< 0.001
**BMI**,** kg/m**^**2**^	29.056 (0.120)	28.819 (0.136)	29.400 (0.136)	0.003
**Sleep duration**,** h/day**	6.905 (0.017)	6.950 (0.026)	6.840 (0.028)	0.011
**Neutrophil count**,** ×10⁹/L**	4.189 (0.031)	4.055 (0.034)	4.384 (0.046)	< 0.001
**Platelet count**,** ×10⁹/L**	236.685 (1.074)	237.611 (1.123)	235.340 (1.603)	0.168
**Monocyte count**,** ×10⁹/L**	0.544 (0.005)	0.528 (0.004)	0.568 (0.008)	< 0.001
**Lymphocyte count**,** ×10⁹/L**	2.053 (0.014)	2.024 (0.017)	2.093 (0.016)	0.001
**Mean PD**,** mm**	1.410 (0.020)	1.138 (0.015)	1.805 (0.022)	< 0.001
**Mean CAL**,** mm**	1.612 (0.031)	1.128 (0.015)	2.315 (0.040)	< 0.001
**Age strata**,** n (%)**				< 0.001
30–44	2675 (35.079)	1735 (42.231)	940 (24.696)	
45–64	3370 (47.196)	1572 (45.059)	1798 (50.297)	
65–80	1629 (17.726)	571 (12.710)	1058 (25.006)	
**Gender**,** n (%)**				< 0.001
Male	3812 (49.623)	1585 (43.432)	2227 (58.609)	
Female	3862 (50.377)	2293 (56.568)	1569 (41.391)	
**Race/ethnicity**,** n (%)**				< 0.001
Non-Hispanic Black	1504 (9.619)	600 (7.194)	904 (13.138)	
Non-Hispanic White	3578 (71.886)	2063 (77.261)	1515 (64.084)	
Mexican American	1074 (7.523)	400 (5.221)	674 (10.864)	
Other Hispanic	750 (4.959)	369 (4.387)	381 (5.790)	
Other Race/multiracial	768 (6.013)	446 (5.937)	322 (6.124)	
**PIR strata**,** n (%)**				< 0.001
<1.3	1933 (15.612)	738 (10.952)	1195 (22.377)	
1.3–3.5	3017 (36.225)	1355 (30.852)	1662 (44.025)	
>3.5	2724 (48.162)	1785 (58.196)	939 (33.598)	
**Marital status**,** n (%)**				< 0.001
Never married	848 (9.547)	444 (9.147)	404 (10.128)	
Married/Living with a partner	5068 (70.806)	2681 (74.626)	2387 (65.261)	
Divorced/Separated/Widowed	1758 (19.646)	753 (16.227)	1005 (24.611)	
**Education level**,** n (%)**				< 0.001
Under high school	1595 (13.388)	512 (8.233)	1083 (20.872)	
High school	1663 (21.004)	694 (17.124)	969 (26.637)	
More than high school	4416 (65.607)	2672 (74.643)	1744 (52.491)	
**Alcohol consumption**,** n (%)**				< 0.001
Never	960 (9.710)	471 (9.484)	489 (10.037)	
Former	1325 (14.630)	528 (11.746)	797 (18.817)	
Mild	2832 (40.154)	1555 (43.189)	1277 (35.748)	
Moderate	1194 (17.938)	712 (19.921)	482 (15.058)	
Heavy	1363 (17.569)	612 (15.660)	751 (20.339)	
**Health insurance**,** n (%)**				< 0.001
Private	4417 (68.033)	2601 (76.338)	1816 (55.978)	
Public	1609 (15.542)	646 (12.229)	963 (20.350)	
Uninsured	1648 (16.425)	631 (11.433)	1017 (23.672)	
**Dental floss/device**,** n (%)**				< 0.001
No	2289 (26.033)	907 (21.449)	1382 (32.688)	
Yes	5385 (73.967)	2971 (78.551)	2414 (67.312)	
**Overall LC9 score**	71.416 (0.283)	73.889 (0.337)	67.826 (0.352)	< 0.001
**Overall LE8 score**	68.819 (0.297)	71.550 (0.356)	64.855 (0.367)	< 0.001
**HEI-2015 diet score**	43.950 (0.632)	45.650 (0.927)	41.481 (0.583)	< 0.001
**Physical activity score**	74.134 (0.747)	75.992 (0.900)	71.437 (1.012)	0.001
**Nicotine exposure score**	74.225 (0.614)	80.871 (0.732)	64.577 (0.857)	< 0.001
**Sleep health score**	83.659 (0.397)	85.245 (0.516)	81.357 (0.495)	< 0.001
**Body mass index score**	59.918 (0.591)	61.227 (0.718)	58.018 (0.758)	0.001
**Blood lipids score**	60.818 (0.497)	62.119 (0.572)	58.929 (0.801)	0.002
**Blood glucose score**	85.081 (0.384)	88.598 (0.453)	79.976 (0.547)	< 0.001
**Blood pressure score**	68.771 (0.563)	72.702 (0.716)	63.065 (0.784)	< 0.001
**PHQ-9 score**	92.189 (0.324)	92.596 (0.405)	91.598 (0.512)	0.129
**ABSI**	0.082 (0.000)	0.081 (0.000)	0.083 (0.000)	< 0.001
**WWI**	11.001 (0.015)	10.900 (0.018)	11.148 (0.017)	< 0.001
**SII**	523.605 (5.772)	508.380 (6.921)	545.704 (8.668)	0.001
**SIRI**	1.219 (0.015)	1.152 (0.017)	1.317 (0.022)	< 0.001

Note: Categorical variables are presented as n (weighted percentage) and continuous variables as mean (SE).

Abbreviations: PIR, poverty-to-income ratio; WC, waist circumference; BMI, body mass index; PD, probing depth; CAL, clinical attachment loss; LC9, Life’s Crucial 9; LE8, Life’s Essential 8; PHQ-9, Patient Health Questionnaire-9; HEI, Healthy Eating Index; SII, Systemic Immune-Inflammation Index; SIRI, System Inflammation Response Index; SE, standard error.

^a^ Chi-square test for categorical variables; T test for continuous variables

### Logistic regression analyses of the associations between LC9 score and periodontitis

Logistic regression models demonstrated an inverse association between elevated LC9 composite score and periodontitis (Table 2**)**. Adjusted regression modeling identified a dose-dependent protective relationship for LC9 composite score against periodontitis, where each 10-point increment corresponded to a 15.5% reduction in disease odds (odds ratio [OR] = 0.845, 95% CI 0.795–0.897; *P* < 0.001). Stratified analyses by LC9 quartiles (reference = Q1) revealed a graded inverse association with periodontitis prevalence in this nationally representative cross-sectional sample, demonstrating sequentially attenuated OR (95% CI) across ascending quartiles: (Q2 = 0.830 (0.703–0.980), *P* = 0.038); Q3 = 0.726 (0.596–0.883), *P* = 0.004); Q4 = 0.620 (0.493–0.780), *P* < 0.001). Trend analysis confirmed a significant and consistent inverse association (*P* for trend < 0.001), indicating progressively reduced periodontitis likelihood with higher LC9 strata in adjusted models. Health behavior components demonstrated a protective association against periodontitis (crude OR = 0.950, 0.943–0.958, *P* < 0.001; adjusted OR = 0.969, 0.961–0.977, *P* < 0.001). Adjusted models further revealed an inverse association between health factors and periodontitis likelihood (OR = 0.985, 0.973–0.996, *P* = 0.017). Conversely, PHQ-9 score exhibited no statistically significant relationship with periodontitis risk after adjustment (OR = 1.033; 0.990–1.078, *P* = 0.142).

**Table 2:** Logistic regression analyses of the associations between life’s crucial 9 score and periodontitis

Variable^a^	Unadjusted model		Adjusted model^b^	
	OR (95%CI)	*P*-value	OR (95%CI)	*P*-value
**LC9**	0.698 (0.663, 0.735)	< 0.001	0.845 (0.795, 0.897)	< 0.001
Q1	Ref.		Ref.	
Q2	0.699 (0.600, 0.815)	< 0.001	0.830 (0.703, 0.980)	0.038
Q3	0.525 (0.449, 0.614)	< 0.001	0.726 (0.596, 0.883)	0.004
Q4	0.313 (0.251, 0.391)	< 0.001	0.620 (0.493, 0.780)	< 0.001
**P for trend**	< 0.001		< 0.001	
**Health behaviours**	0.950 (0.943, 0.958)	< 0.001	0.969 (0.961, 0.977)	< 0.001
**Health factors**	0.953 (0.944, 0.962)	< 0.001	0.985 (0.973, 0.996)	0.017
**PHQ9 score**	0.970 (0.934, 1.008)	0.128	1.033 (0.990, 1.078)	0.142
**HEI-2015 diet score**	0.959 (0.940, 0.979)	< 0.001	0.981 (0.961, 1.002)	0.085
**Physical activity score**	0.973 (0.959, 0.987)	< 0.001	0.994 (0.979, 1.008)	0.393
**Nicotine exposure score**	0.886 (0.871, 0.901)	< 0.001	0.901 (0.886, 0.917)	< 0.001
**Sleep health score**	0.934 (0.911, 0.958)	< 0.001	0.974 (0.947, 1.001)	0.073
**Body mass index score**	0.970 (0.954, 0.987)	0.001	0.991 (0.972, 1.010)	0.374
**Blood lipids score**	0.965 (0.946, 0.986)	0.002	0.990 (0.965, 1.016)	0.463
**Blood glucose score**	0.864 (0.844, 0.884)	< 0.001	0.946 (0.921, 0.971)	< 0.001
**Blood pressure score**	0.901 (0.882, 0.921)	< 0.001	0.972 (0.948, 0.996)	0.032

^a^Per 10-score increase

^b^Adjusted for age, gender, race/ethnicity, PIR, marital status, education level, alcohol consumption, health insurance and dental floss/device.

Abbreviations: LC9, Life’s Crucial 9; OR, odds ratio; CI, confidence interval; PHQ-9, Patient Health Questionnaire-9; HEI, Healthy Eating Index; PIR, poverty-to-income ratio

### RCS and threshold effect analysis of LC9 score with periodontitis

**Fig. 3:**
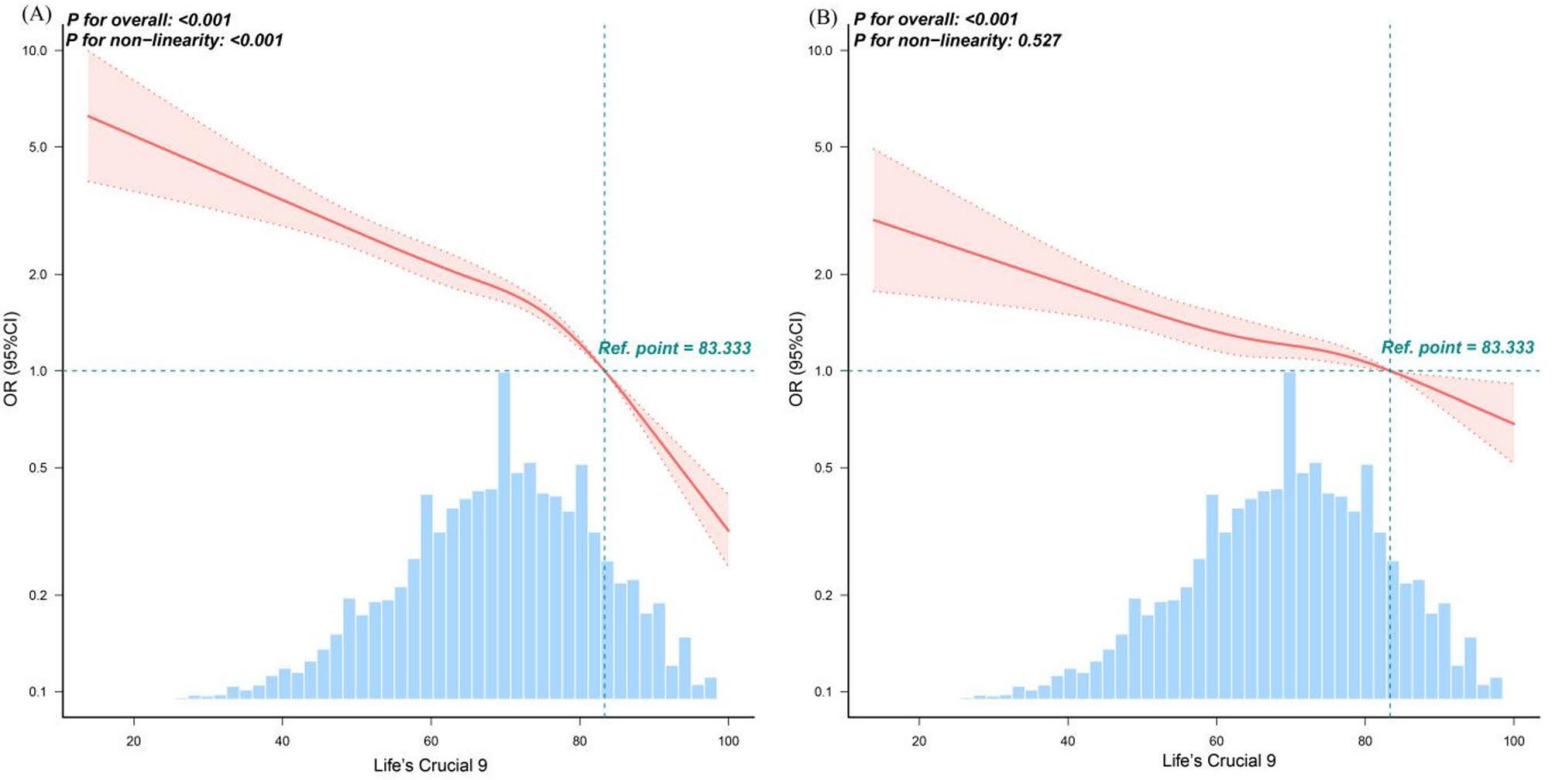
Dose–response relationships between Life’s Crucial 9 and periodontitis. (**A**) Unadjusted; (**B**) Adjusted for age, gender, race/ethnicity, PIR, marital status, education level, alcohol consumption, health insurance and dental floss/device. ORs (solid lines) and 95% confidence levels (dotted lines). Abbreviations: OR, odds ratio; CI, confidence interval; LC9, Life’s Crucial 9

RCS analysis in the unadjusted model revealed a significant nonlinear inverse relationship between LC9 score and periodontitis prevalence (*P* for nonlinear < 0.001), with a turning point at LC9 = 83.333. Below this threshold, each 1-point increase in LC9 was associated with a 2.5% reduction in periodontitis odds (OR = 0.975; 95% CI: 0.971–0.979; *P* < 0.001), while above the threshold, the protective effect strengthened (OR = 0.905; 95% CI: 0.885–0.926; *P* < 0.001). However, in fully adjusted models, the association became linear (*P* for nonlinear = 0.527), as shown in Fig. 3 and Supplementary Table S3.

### Subgroup analysis for the association between LC9 and periodontitis

**Fig. 4:**
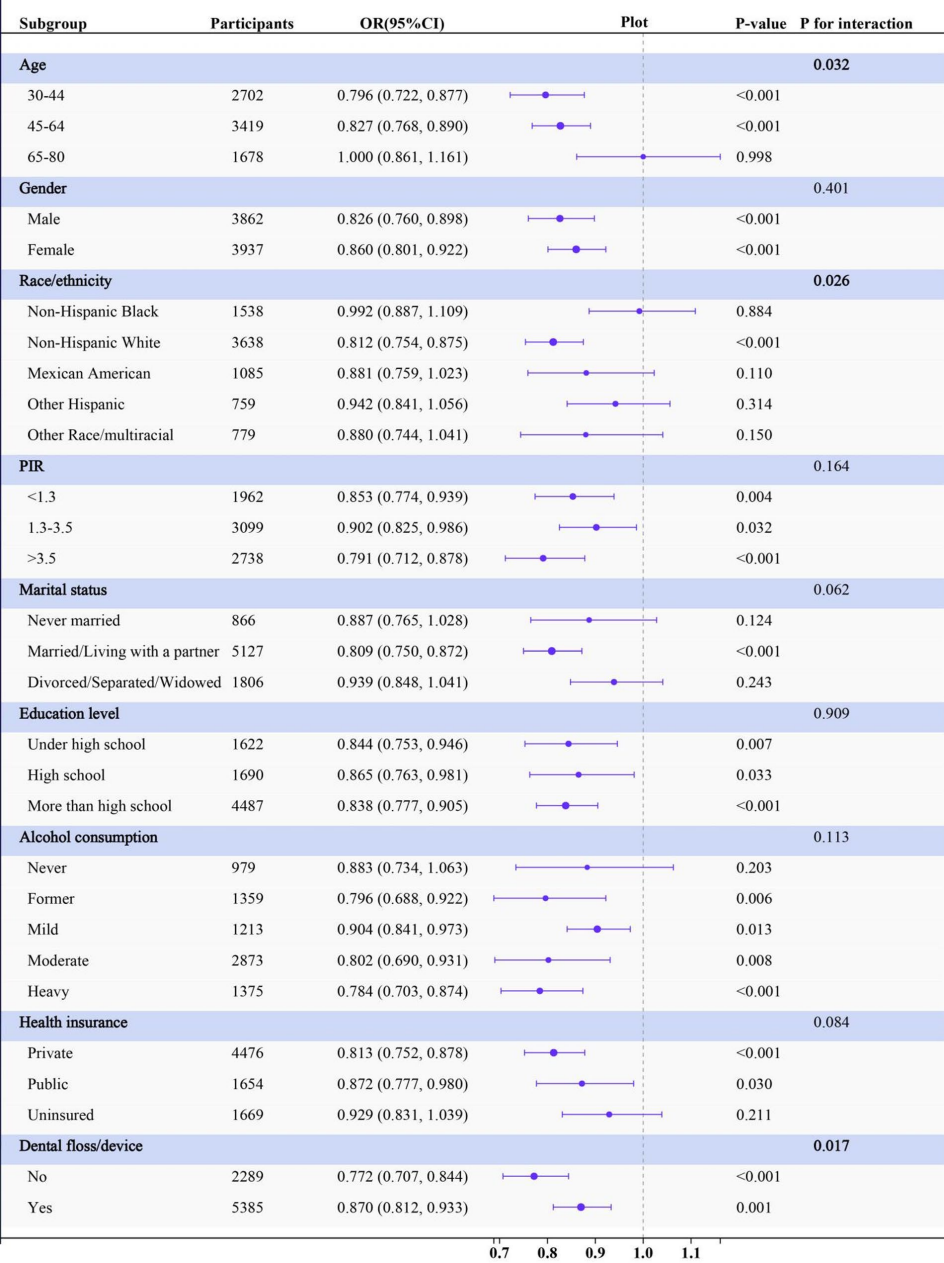
Subgroup analysis for the associations between per Life’s Crucial 9 10-score increase and periodontitis. The multivariable model is adjusted for age, gender, race/ethnicity, PIR, marital status, education level, alcohol consumption, health insurance and dental floss/device except the corresponding stratification variable. Abbreviations: OR, odds ratio; CI, confidence interval; PIR, poverty-to-income ratio

Age demonstrated a significant interaction effect (*P* for interaction = 0.032), with individuals aged 30–44 reducing periodontitis odds by 20.4% (OR, 0.796, 95% CI: 0.722–0.877) and individuals aged 45–64 reducing periodontitis odds by 17.3% (OR, 0.827, 95% CI: 0.768–0.890), although no significant difference was observed in the 65–80 years group (*P* = 0.998). Race also demonstrated a significant interaction effect (*P* for interaction = 0.026). Non-Hispanic White individuals exhibited significant association, with decreasing of periodontitis odds by 18.8% (OR, 0.812, 95%CI: 0.754–0.875). Significant effect modification by flossing was observed (*P*-interaction = 0.017). Non-flossers showed stronger LC9 protection (OR = 0.772, 95%CI: 0.707–0.844) than flossers (OR = 0.870, 95%CI: 0.812–0.933). Evaluation of interaction effects demonstrated no statistically significant modification by other subgroups **(**Fig. 4**)**.

### Association between components of the LC9 and periodontitis

**Fig. 5:**
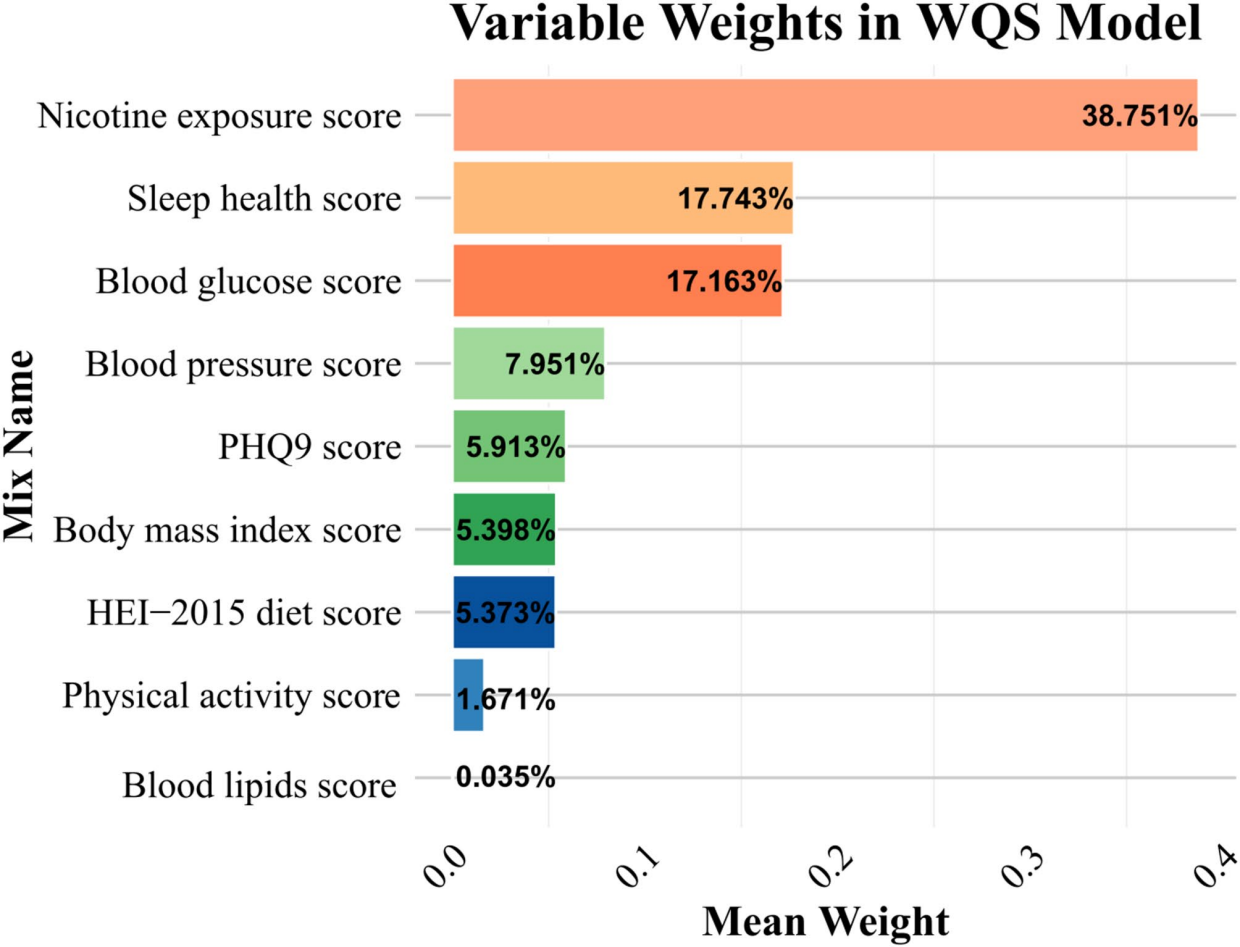
WQS analysis of LC9 metrics and Periodontitis. Models were adjusted for age, gender, race/ethnicity, PIR, marital status, education level, alcohol consumption, health insurance and dental floss/device. Abbreviations: WQS, Weighted Quantile Sum, PHQ-9, Patient Health Questionnaire-9; HEI, Healthy Eating Index; LC9, Life’s Crucial 9; PIR, poverty-to-income ratio

WQS regression analyses identified an association between the nine composite variables and periodontitis risk. While WQS does not estimate the direct association between the overall LC9 score and periodontitis, it quantifies the weighted contribution of each component within a composite exposure model. The results revealed nicotine exposure (38.751%), sleep health (17.743%), blood glucose (17.163%), blood pressure (7.951%) and PHQ-9 (5.913%) as dominant contributors to this protective association, as visually summarized in Fig. 5.

Multivariable logistic regression analysis also evaluated the association between LC9 metrics and periodontitis. Significant inverse associations were observed for nicotine exposure (OR = 0.884, 95% CI: 0.867–0.900, *P* < 0.001), blood glucose (OR = 0.884, 95% CI: 0.865–0.903, *P* < 0.001), and blood pressure (OR = 0.915, 95% CI: 0.893–0.937, *P* < 0.001). Sleep health also showed a modest inverse association (OR = 0.956, 95% CI: 0.928–0.985, *P* = 0.005). Conversely, higher PHQ-9 score was associated with increased odds of periodontitis (OR = 1.049, 95% CI: 1.007–1.092, *P* = 0.026) (Supplementary Table S4).

### Predictive ability of LC9 compared to LE8 for periodontitis

**Fig. 6:**
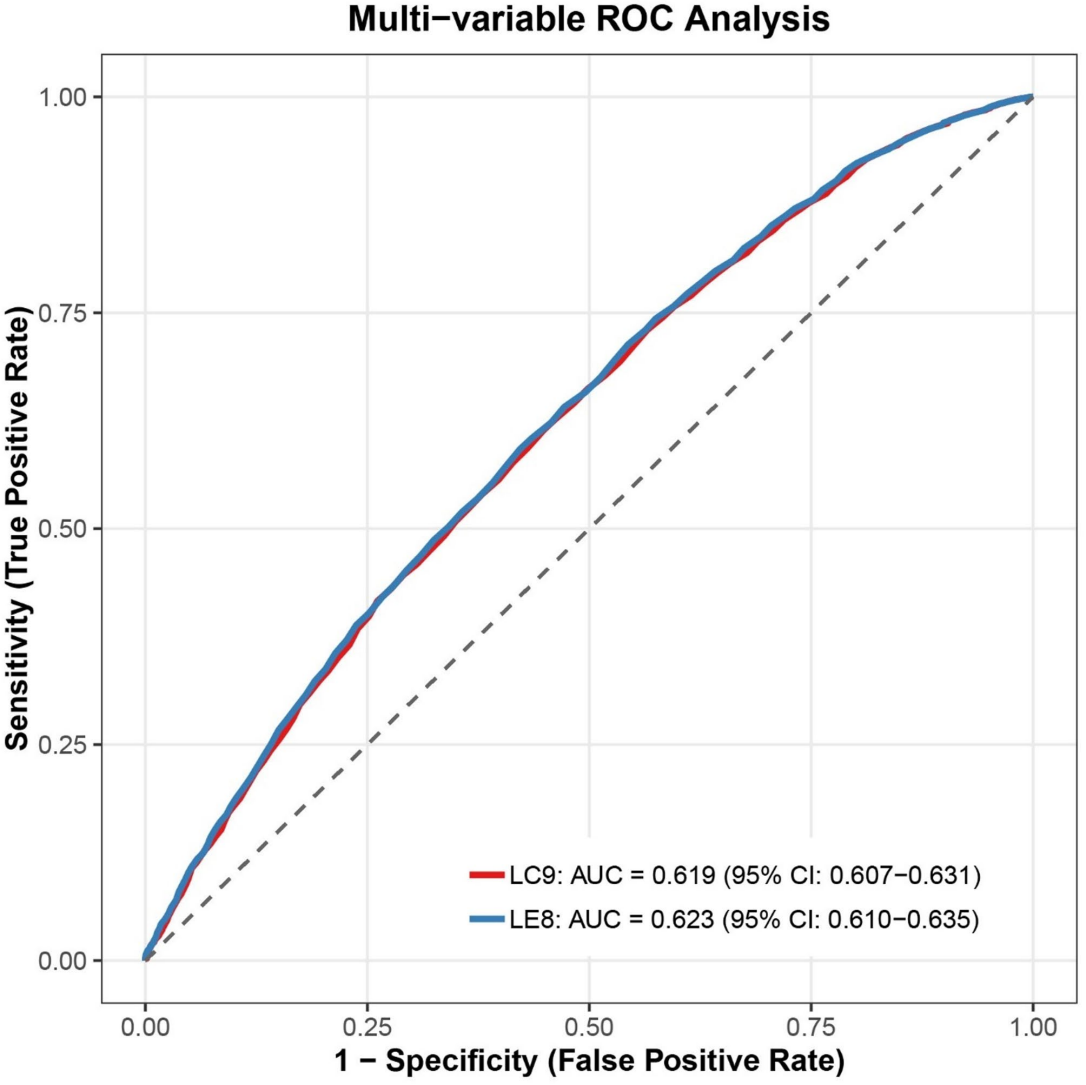
ROC curve for predictive ability of LC9 compared to LE8 for periodontitis

ROC analysis revealed comparable diagnostic accuracy between LC9 and LE8 for periodontitis detection, with LC9 showing marginally lower discriminative ability (AUC: 0.619 vs. 0.623; Fig. 6).

### Sensitivity analyses

To verify the robustness of the findings, three complementary sensitivity analyses were conducted. First, multinomial logistic regression confirmed a dose-dependent inverse association between LC9 quartiles and both non-severe and severe periodontitis, consistent with the main binary logistic models (Supplementary Table S5). Each 10-point increase in LC9 score was associated with 25.7% and 35.7% lower odds of non-severe and severe periodontitis, respectively. Similar protective trends were observed for composite health behaviors and factors, while PHQ-9 scores showed no independent association.

Second, multivariable linear regressions demonstrated that higher LC9 scores were significantly associated with reduced periodontal PD and CAL. Specifically, each 10-point LC9 increment corresponded to a 0.097 mm decrease in mean PD and a 0.181 mm decrease in mean CAL (Supplementary Table S6).

Third, complete-case analyses excluding participants with missing covariates yielded results comparable to the primary models, affirming the inverse association between LC9 and periodontitis risk (adjusted OR = 0.850; 95% CI: 0.800–0.903; *P* < 0.001). Among individual LC9 components, nicotine avoidance and blood glucose control remained significant contributors (Supplementary Table S7).

Finally, in this additional sensitivity analysis using the dietary recall subsample weight (WTDRD1/3), the inverse association between LC9 scores and periodontitis remained consistent with the results obtained using WTMEC2YR/3 (Supplementary Table S8). Effect estimates and confidence intervals showed minimal variation, confirming that the relationship between LC9 and periodontitis is robust to alternative NHANES weighting approaches. This further reinforces the validity of the observed association, particularly in relation to dietary components of the LC9 score.

### Mediation analyses

To explore potential pathways linking LC9 and periodontitis, mediation analyses were conducted using central adiposity indices (ABSI, WWI) and inflammatory biomarkers (SII, SIRI) as candidate mediators. Preliminary weighted logistic and linear regression confirmed that all four variables were significantly associated with both LC9 scores and periodontitis risk (Supplementary Tables S9–S10).

**Fig. 7:**
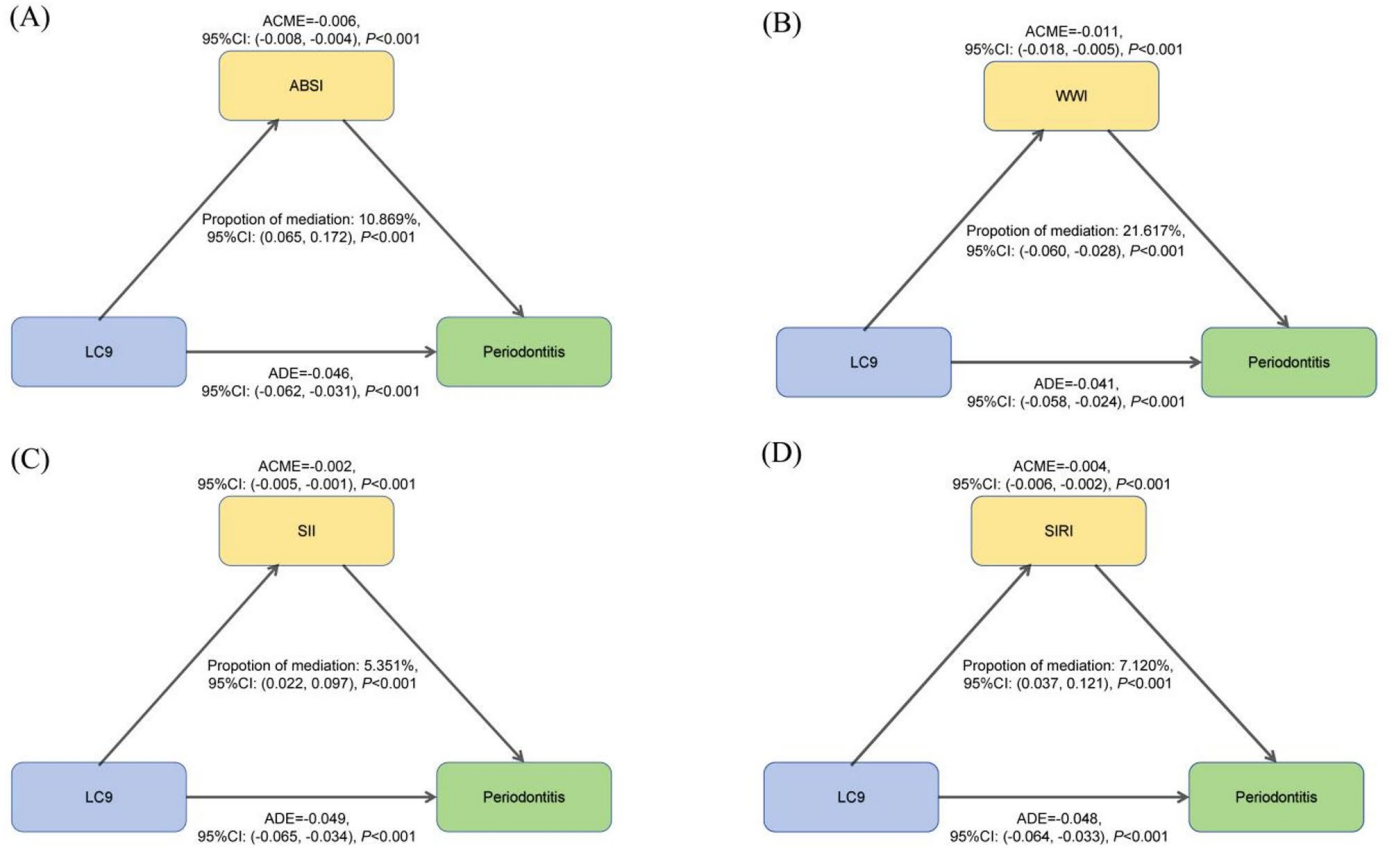
Estimated proportion of the association between LC9 and periodontitis mediated by ABSI (**A**), WWI (**B**), SII (**C**) and SIRI (**D**). Models were adjusted for age, gender, race/ethnicity, PIR, marital status, education level, alcohol consumption, health insurance and dental floss/device. Abbreviations: CI, confidence interval; LC9, Life’s Crucial 9; ABSI, a body shape index; WWI, weight-adjusted-waist index; SII, Systemic Immune-Inflammation Index; SIRI, System Inflammation Response Index; ACME, average causal mediation effect; ADE, average direct effect

Formal mediation analyses under the counterfactual framework revealed significant indirect effects for all mediators (all *P* < 0.001; Fig. 7). WWI contributed the largest proportion of the total effect (21.617%), followed by ABSI (10.869%), SIRI (7.120%), and SII (5.351%), indicating that both central adiposity and systemic inflammation partially mediate the protective relationship between LC9 and periodontitis, with WWI being the most influential mediator.

## Discussion

This cross-sectional analysis is the first to examine the association between Life’s Crucial 9 (LC9)—a novel cardiovascular health metric incorporating psychological well-being—and periodontitis prevalence. Consistent with previous findings linking LE8 scores to lower periodontitis risk [31, 32], our study extends this evidence by highlighting the inverse association between higher LC9 scores and periodontitis, with behavioral components such as nicotine exposure (38.751%), sleep health (17.743%), blood glucose control (17.163%), blood pressure (7.951%) and PHQ-9 (5.913%) contributing most prominently. Stratified analyses identified effect modification by age, race, and flossing behavior, with stronger protective effects in younger adults, non-Hispanic Whites, and non-flossers. Mediation analyses further demonstrated that both central adiposity and systemic inflammation partially explain this relationship, aligning with evidence that visceral fat–driven cytokines (e.g., IL-6, TNF-α) contribute to systemic inflammation involved in both cardiovascular and periodontal diseases [33, 34]. Overall, a 10-unit increase in LC9 score was associated with a 15.5% reduction in the odds of periodontitis, underscoring the relevance of holistic cardiovascular and behavioral health in periodontal disease prevention.

After adjusting for confounders, WQS analysis identified nicotine exposure, blood pressure, blood glucose, sleep quality, and psychological health as significant contributors to periodontitis, aligning with the main findings and highlighting systemic inflammation as a central mechanistic pathway [35]. Chronic low-grade inflammation promotes sustained cytokine production and activation of inflammatory cascades, contributing to both cardiometabolic and periodontal diseases [36]. Neutrophil-driven responses, particularly involving S100A12, have been implicated in the pathogenesis of periodontitis by mediating both local and systemic inflammatory processes [37, 38]. These immune-inflammatory mechanisms are closely linked to key LC9 components.

Among these, nicotine exposure exerted the strongest influence (38.751%), likely through mechanisms involving oxidative stress, immune dysregulation, and promotion of central adiposity [39–41]. Elevated blood glucose (17.163%) and blood pressure (7.951%) may impair tissue repair, alter the oral microbiota, and enhance inflammatory responses, thereby aggravating periodontal damage [42–44]. Notably, sleep health (17.743%) emerged as a substantial factor, possibly through its regulatory role in circadian rhythm, cortisol levels, and inflammatory cytokine release [45–47]. These findings underscore the importance of behavioral risk factors and systemic inflammation—particularly central adiposity-related pathways—in mediating the LC9–periodontitis association, providing mechanistic insights for prevention and intervention strategies.

Psychological health, though contributing a smaller proportion (5.913%) to periodontitis risk, may still play a meaningful role through its complex interplay with systemic inflammation and immune dysregulation. Midlife central adiposity has been linked to elevated risks of depressive disorders later in life [48], while chronic psychological stress alters neuroendocrine function, elevating cortisol levels and suppressing immune responses against periodontal pathogens [49]. Psychiatric conditions are also associated with increased pro-inflammatory cytokines such as IL-6 and TNF-α, exacerbating systemic inflammation and periodontal destruction [50]. Additionally, oxidative stress in mental disorders may impair tissue repair and promote osteoclast activity through ROS-mediated pathways [51, 52].

Although prior studies have reported associations between depression and periodontitis [53], our regression analysis did not identify a significant link between PHQ-9 scores and periodontitis, consistent with findings from Mendelian randomization and NHANES-based research [54, 55]. Nevertheless, WQS analysis suggested a potential contribution, indicating the need for caution in interpreting null associations. This discrepancy may reflect limitations of cross-sectional data, cultural differences, or the overlapping effects of depression with other LC9 components. Moreover, reliance on the PHQ-9 may underestimate the role of psychological health due to its subjective nature, limited diagnostic scope, and insufficient assessment of symptom severity [4]. These findings highlight the necessity for more robust, objective mental health measures to better capture psychological influences on periodontal outcomes within the LC9 framework.

Body mass index (BMI), HEI-2015 diet score, and physical activity were identified as notable contributors to periodontitis risk, accounting for 5.398%, 5.373%, and 1.671% of the association, respectively. Elevated BMI may exacerbate periodontal inflammation through obesity-induced metabolic dysregulation and systemic inflammation, with genetic evidence suggesting a 15% increased risk of periodontitis per standard deviation increase in BMI [56, 57]. In contrast, low BMI may heighten vulnerability through impaired immune responses and nutritional deficiencies [58]. A higher HEI-2015 score appears protective, potentially due to the modulation of the oral and gut microbiota by anti-inflammatory nutrients [59]. In this context, natural substances such as probiotics, postbiotics, postbiotics, and ozonized compounds have shown promise in supporting long-term microbial eubiosis and may serve as adjunctive dietary components to further enhance periodontal resilience [60, 61]. Physical activity, particularly when of higher intensity, may contribute additional benefit by improving metabolic health and dampening systemic inflammation [62].

Subgroup analysis revealed a link between LC9 score and periodontitis in individuals under 65, suggesting that age may influence the association between periodontitis and cardiovascular disease [63]. Non-Hispanic White individuals were at higher risk for periodontitis [18], likely influenced by genetic and lifestyle factors [64], as well as a greater susceptibility to heart disease [65]. However, severe periodontitis prevalence was higher among Hispanic and non-Hispanic Black adults [66], potentially due to socioeconomic disparities and access to healthcare, which influence systemic inflammation and overall health [67]. The observed difference likely reflects floss users’ baseline protection from better oral hygiene, attenuating LC9’s effect, with potential contributions from behavioral and socioeconomic factors [68, 69].

In this study, ROC curve analysis indicated that LC9 and LE8 exhibited similar diagnostic performance in identifying periodontitis, with LC9 displaying a slightly lower AUC (0.619 vs. 0.623). Nonetheless, the significance of these metrics likely extends beyond prediction accuracy. By integrating both cardiovascular and psychological dimensions, LC9 offers a more holistic view of health status relevant to periodontal disease. This expanded framework may prove especially valuable in complex or high-risk subgroups, such as those with mental health conditions. Future studies should investigate the cost-effectiveness and real-world applicability of LC9 and LE8 in varied clinical settings to support broader implementation.

Future longitudinal studies are essential to both confirm the predictive relevance of LC9 for periodontitis and to clarify the underlying biological and behavioral mechanisms. If validated, LC9 could be utilized as a practical tool in routine dental assessments to identify individuals with suboptimal cardiovascular health, enabling early, tailored interventions that may improve both oral and systemic outcomes. Emphasis should be placed on modifiable risk factors, such as BMI and sleep patterns, through strategies like personalized diet plans, structured physical activity, and sleep hygiene education, aligned with an individual’s LC9 profile.

Moreover, the current cardiovascular health frameworks, including LC9, still inadequately incorporate psychological domains. Limitations in NHANES—particularly in the subjective nature of PHQ-9—highlight the urgent need for more robust and objective measures of mental health. Future research should explore the integration of broader psychosocial factors, such as resilience, stress adaptation, and social support, into CVH scoring systems. These additions may enhance the model’s predictive performance not only for periodontitis but also for comprehensive health outcomes.

## Strengths and limitations

This study offers several notable strengths. To the best of our knowledge, it is the first to explore the relationship between LC9—a newly proposed cardiovascular health construct—and the prevalence of periodontitis, while also investigating the intermediary roles of central adiposity markers (ABSI and WWI) and systemic inflammatory indices (SII and SIRI) within a large, nationally representative sample of U.S. adults. The integrative framework of LC9, which encompasses psychosocial well-being, lifestyle behaviors, and metabolic parameters, facilitates a more holistic evaluation of cardiovascular–oral health interconnections. By incorporating NHANES sampling weights, our analysis provides population-level insights and identifies a negative association between LC9 scores and periodontitis risk. These results carry important implications for public health strategies, emphasizing the potential of behavioral interventions aimed at enhancing LC9 metrics to reduce periodontal disease burden. Furthermore, the identified mediating roles of abdominal adiposity and inflammation offer mechanistic insights and may assist in recognizing vulnerable populations for targeted prevention and early management.

Despite the strengths of utilizing a nationally representative sample and incorporating a novel cardiovascular health construct, this study is inherently limited by its cross-sectional design, which precludes definitive conclusions about causality. Furthermore, several LC9 components—such as dietary habits, physical activity, tobacco exposure, sleep quality, and depressive symptoms—were based on self-reported data, introducing potential recall bias and subjective variability. The NHANES dataset also lacks information on other relevant psychological constructs, including stress levels and anxiety, which may further influence periodontal outcomes. Future longitudinal studies are essential to elucidate the temporal and biological pathways connecting LC9 to periodontitis.

## Conclusions

This study identifies an inverse relationship between LC9 scores and periodontitis prevalence and severity, partially mediated by central adiposity and systemic inflammation. Key LC9 components—including nicotine exposure, sleep, glycemic control, blood pressure, and depressive symptoms—were closely linked to periodontal status. Although LC9 did not outperform LE8 in predictive value, its multidimensional nature highlights potential targets for intervention. Future longitudinal studies should assess LC9’s utility in broader clinical contexts. Meanwhile, implementing lifestyle interventions targeting modifiable LC9 components—such as promoting smoking cessation, optimizing sleep, and addressing metabolic and psychological health—may serve as practical strategies to reduce periodontitis risk and improve overall oral health.

## Electronic supplementary material

Below is the link to the electronic supplementary material.

Supplementary Material 1

## Data Availability

Data were utilized from the official NHANES website (https://wwwn.cdc.gov/Nchs/Nhanes/).

## Abbreviations

LC9: Life’s Crucial 9
LE8: Life’s Essential 8
CVH: Cardiovascular health
NHANES: National Health and Nutrition Examination Survey
AHA: American Heart Association
AAP: American Academy of Periodontology
WC: Waist circumference
WHtR: Waist-to-Height Ratio
ABSI: A body shape index
WWI: Weight-adjusted-waist index
SII: Systemic immune inflammation index
SIRI: System inflammation response index
CVD: Cardiovascular diseases
BMI: Body mass index
NCHS: National Center for Health Statistics
RERB: Research Ethics Review Board
PIR: Poverty-to-income ratio
CDC: Centers for Disease Control and Prevention
PD: Pocket depth
CAL: Clinical attachment loss
SE: Standard error
WQS: Weighted Quantile Sum
PHQ-9: Patient Health Questionnaire-9
OR: Odds ratio
CI: Confidence interval
ADE: Average direct effect
MP: Mediation proportion
ACME: Average causal mediation effect
IL-6: Interleukin-6
CRP: C-reactive protein
TNF-α: Tumor necrosis factor-α
