# Supplementary material for: Central adiposity indices and inflammatory markers mediate the association between life’s crucial 9 and periodontitis in US adults
Source: Lipids Health Dis. 2025 Jun 3;24:199. doi: 10.1186/s12944-025-02619-1 (PMC12131473; doi:10.1186/s12944-025-02619-1)
Supplement: Supplementary file 1 — Supplementary Material 1 [file 12944_2025_2619_MOESM1_ESM.docx]

**Supplementary Material**

**Text S1.** Periodontal examination and Definitions.

Participants aged 30 years and older were eligible for periodontal exams if they had at least one tooth and no medical exclusions. Periodontitis data were derived from Oral Health-Periodontal in Examination data in NHANES 2009–2014. Examiners measured the gingival recession (REC) and pocket depth (PD) at six sites on each tooth using a periodontal probe (2-mm increments, HU-Friedy). All measurements were rounded to the lowest whole millimetre. The NHANES Integrated Survey and Information System (ISIS) computed clinical attachment loss (CAL) as the difference between PD and REC.

Periodontitis prevalence was assessed using the CDC/American Academy of Periodontology (AAP) case definitions. Severe periodontitis was defined as ≥2 interproximal sites with CAL ≥ 6 mm (not on same tooth), and ≥1 interproximal sites with PD ≥ 5 mm. Moderate periodontitis was defined as ≥2 interproximal sites with CAL ≥ 4 mm (not on same tooth), or ≥2 interproximal sites with PD ≥ 5 mm (not on same tooth). Mild periodontitis was defined as ≥2 interproximal sites with CAL ≥ 3 mm, and ≥2 interproximal sites with PD ≥ 4 mm (not on same tooth) or one site with PD ≥ 5 mm. For this study and in accordance with prior literature, we used the presence of periodontitis, which we dichotomized as ‘Yes’ (i.e., presence of mild, moderate or severe periodontitis) or ‘No’ as our primary outcome. Additionally, mean PD and mean CAL was calculated as secondary outcomes for periodontal disease.

**Text S2.** Definition of variables involved in this study.

Based on previous research and clinical practice, demographic characteristics were obtained from the household interview questionnaires, and for this study, gender (male, female), age (30–44 years old, 45–64 years old, and 65–80 years old), race (Mexican American, non-Hispanic black, non-Hispanic white, other Hispanic, and other races), marital status (never married, married/living with a partner, and divorced/separated/widowed), education level (under high school, high school, and more than high school), poverty-to-income ratio (PIR) (<1.3, 1.3-3.5 and>3.5), alcohol use (heavy, moderate, mild, former and never) were used as covariates, health insurance (private, public and uninsured) and use of dental floss/device (no and yes) .

**Table S1.** Definition and scoring approach for the Life’s Crucial 9 score.

| Domain | CVH Metric | Measurement | Quantification and Scoring of CVH Metric |
| --- | --- | --- | --- |
| Psychological  Health | Depressive symptoms | Depression Screener Questionnaire (DPQ) | Metric: Nine-item depression screening instrument PHQ-9. Each symptom item in PHQ-9 is scored on a 4-point scale, from 0 (‘not at all’) to 3 (‘nearly every day’), resulting in a total score of 0 to 27 points.  Scoring:  Points Level  100 the score of 0 to 4 points  70 the score of 5 to 9 points  30 the score of 10 to 14 points  0 the score of 15 to 27 points |
| Health Behaviors | Diet | Healthy Eating Index-2015 diet score percentile | Quantiles of DASH-style diet adherence  Scoring (Population):  Points Quantile  100 ≥95th percentile (top/ideal diet)  80 75th – 94th percentile  50 50th – 74th percentile  25 25th – 49th percentile  0 1st – 24th percentile (bottom/least ideal quartile) |
|  | Physical activity | Self-reported minutes of moderate or vigorous physical activity per week | Metric: Minutes of moderate (or greater) intensity activity per week  Scoring:  Points Minutes  100 ≥150  90 120 – 149  80 90 – 119  60 60 – 89  40 30 – 59  20 1 – 29  0 0 |
|  | Nicotine exposure | Self-reported use of cigarettes or inhaled nicotine-delivery system | Metric: Combustible tobacco use and inhaled NDS use; or secondhand smoke exposure  Scoring:  Points Status  100 Never smoker  75 Former smoker, quit ≥5 yrs  50 Former smoker, quit 1 - <5 yrs  25 Former smoker, quit <1 year, or currently using inhaled NDS  0 Current smoker  Subtract 20 points (unless the score is 0) for living with an active indoor smoker in the home |
|  | Sleep health | Self-reported average hours of sleep per night | Metric: Average hours of sleep per night  Scoring:  Points Level  100 7 – <9  90 9 – <10  70 6 – <7  40 5 – <6 or ≥10  20 4 – <5  0 <4 |
| Health Factors | Body mass index | Body weight (kg) divided by height squared (m2) | Metric: Body mass index (kg/m2)  Scoring:  Points Level  100 <25.0  70 25.0 – 29.9  30 30.0 – 34.9  15 35.0 – 39.9  0 ≥40.0 |
|  | Blood lipids | Plasma total and HDL-cholesterol with the calculation of non-HDL-cholesterol | Metric: Non-HDL-cholesterol (mg/dL)  Scoring:  Points Level  100 <130  60 130 – 159  40 160 – 189  20 190 – 219  0 ≥220  If the drug-treated level, subtract 20 points |
|  | Blood glucose | Fasting blood glucose or casual hemoglobin A1c | Metric: Fasting blood glucose (mg/dL) or Hemoglobin A1c (%)  Scoring:  Points Level  100 No history of diabetes and FBG <100 (or HbA1c < 5.7)  60 No diabetes and FBG 100 – 125 (or HbA1c 5.7-6.4) (Pre-diabetes)  40 Diabetes with HbA1c <7.0  30 Diabetes with HbA1c 7.0 – 7.9  20 Diabetes with HbA1c 8.0 – 8.9  10 Diabetes with Hb A1c 9.0 – 9.9  0 Diabetes with HbA1c ≥10.0 |
|  | Blood pressure | Appropriately measured systolic and diastolic blood pressure | Metric: Systolic and diastolic blood pressure (mm Hg)  Scoring:  Points Level  100 <120/<80 (Optimal)  75 120-129/<80 (Elevated)  50 130-139 or 80-89 (Stage I HTN)  25 140-159 or 90-99  0 ≥160 or ≥100  Subtract 20 points if treated level |

**Table S2.** Healthy Eating Index-2015 Components & Scoring Standards.

| **Component** | **Maximum points**^1^ | **The standard for maximum score** | **The standard for a minimum score of zero** |
| --- | --- | --- | --- |
| ***Adequacy*** | | | |
| Total Fruits^[2](https://epi.grants.cancer.gov/hei/developing.html#f2)^ | 5 | ≥0.8 cup equiv. per 1,000 kcal | No Fruit |
| Whole Fruits^[3](https://epi.grants.cancer.gov/hei/developing.html#f3)^ | 5 | ≥0.4 cup equiv. per 1,000 kcal | No Whole Fruit |
| Total Vegetables^[4](https://epi.grants.cancer.gov/hei/developing.html#f4)^ | 5 | ≥1.1 cup equiv. per 1,000 kcal | No Vegetables |
| Greens and Beans^[4](https://epi.grants.cancer.gov/hei/developing.html#f4)^ | 5 | ≥0.2 cup equiv. per 1,000 kcal | No Dark Green Vegetables or Legumes |
| Whole Grains | 10 | ≥1.5 oz equiv. per 1,000 kcal | No Whole Grains |
| Dairy^[5](https://epi.grants.cancer.gov/hei/developing.html#f5)^ | 10 | ≥1.3 cup equiv. per 1,000 kcal | No Dairy |
| Total Protein Foods^[6](https://epi.grants.cancer.gov/hei/developing.html#f6)^ | 5 | ≥2.5 oz equiv. per 1,000 kcal | No Protein Foods |
| Seafood and Plant Proteins^[6](https://epi.grants.cancer.gov/hei/developing.html#f6),[7](https://epi.grants.cancer.gov/hei/developing.html#f7)^ | 5 | ≥0.8 oz equiv. per 1,000 kcal | No Seafood or Plant Proteins |
| Fatty Acids^[8](https://epi.grants.cancer.gov/hei/developing.html#f8)^ | 10 | (PUFAs + MUFAs)/SFAs ≥2.5 | (PUFAs + MUFAs)/SFAs ≤1.2 |
| ***Moderation*** | | | |
| Refined Grains | 10 | ≤1.8 oz equiv. per 1,000 kcal | ≥4.3 oz equiv. per 1,000 kcal |
| Sodium | 10 | ≤1.1 gram per 1,000 kcal | ≥2.0 grams per 1,000 kcal |
| Added Sugars | 10 | ≤6.5% of energy | ≥26% of energy |
| Saturated Fats | 10 | ≤8% of energy | ≥16% of energy |

(1) Intakes between the minimum and maximum standards are scored proportionately.

(2) Includes 100% fruit juice.

(3) Includes all forms except juice.

(4) Includes legumes (beans and peas).

(5) Includes all milk products, such as fluid milk, yogurt, cheese, and fortified soy beverages.

(6) Includes legumes (beans and peas).

(7) Includes seafood, nuts, seeds, soy products (other than beverages), and legumes (beans and peas).

(8) Ratio of poly- and monounsaturated fatty acids (PUFAs and MUFAs) to saturated fatty acids (SFAs).

Adequacy components represent the food groups, subgroups, and dietary elements that are encouraged. Higher scores reflect higher intakes for these components because higher intakes are desirable.

Moderation components represent the food groups and dietary elements for which there are recommended limits to consumption. For moderation components, higher scores reflect lower intakes, because lower intakes are more desirable.

**Table S3.**Threshold effect analysis of Life's Crucial 9 score with periodontitis.

| **Outcome:periodontitis** | **OR (95%CI) P-value** |
| --- | --- |
| **Model I** |  |
| **One line effect** | 0.968 (0.964, 0.971) <0.001 |
| **Model II** |  |
| **Turning point(K)** | 83.333 |
| **< K effect 1** | 0.975 (0.971, 0.979) <0.001 |
| **> K effect 2** | 0.905 (0.885, 0.926) <0.001 |
| **LRT test** | <0.001 |
| Abbreviations:OR, odds ratio; CI, confidence interval; LRT, log-likelihood ratio test | |

**Table S4.** Multivariable logistic regression analyses of the association between individual Life's Crucial 9 metrics and periodontitis

| **Outcome:Periodontitis** | **OR (95%CI)** | **P-value** |
| --- | --- | --- |
| **HEI‐2015 diet score** | 0.989 (0.975, 1.003) | 0.122 |
| **Physical activity score** | 0.988 (0.968, 1.007) | 0.223 |
| **Nicotine exposure score** | 0.884 (0.867, 0.900) | **<0.001** |
| **Sleep health score** | 0.956 (0.928, 0.985) | **0.005** |
| **Body mass index score** | 1.009 (0.994, 1.024) | 0.265 |
| **Blood lipids score** | 0.988 (0.968, 1.008) | 0.252 |
| **Blood glucose score** | 0.884 (0.865, 0.903) | **<0.001** |
| **Blood pressure score** | 0.915 (0.893, 0.937) | **<0.001** |
| **PHQ-9score** | 1.049 (1.007, 1.092) | **0.026** |

**Table S5.** Multinomial logistic regression analyses of the associations between Life's Crucial 9 score and periodontitis.

| **Variable^a^** | **Nonsevere periodontitis^b^** | | **Severe periodontitisb** | |
| --- | --- | --- | --- | --- |
|  | **OR (95%CI)^c^** | **P-value** | **OR (95%CI)^c^** | **P-value** |
| **LC9** | 0.743 (0.716,0.772) | <0.001 | 0.643 (0.606, 0.682) | <0.001 |
| Q1 | Ref. | | Ref. | |
| Q2 | 0.705 (0.615,0.809) | <0.001 | 0.663 (0.542, 0.811) | <0.001 |
| Q3 | 0.578 (0.505,0.663) | <0.001 | 0.455 (0.368, 0.561) | <0.001 |
| Q4 | 0.378 (0.329,0.434) | <0.001 | 0.190 (0.148, 0.245) | <0.001 |
| **P for trend** | <0.001 |  | <0.001 |  |
| **Health behaviours** | 0.964 (0.958,0.970) | <0.001 | 0.935 (0.926, 0.945) | <0.001 |
| **Health factors** | 0.956 (0.950,0.963) | <0.001 | 0.950 (0.940, 0.960) | <0.001 |
| **PHQ-9 score** | 0.989 (0.965,1.013) | 0.368 | 0.970 (0.933, 1.008) | 0.116 |
| **HEI‐2015 diet score** | 0.971 (0.956,0.985) | <0.001 | 0.940 (0.917, 0.963) | <0.001 |
| **Physical activity score** | 0.972 (0.962,0.983) | <0.001 | 0.985 (0.967, 1.003) | 0.102 |
| **Nicotine exposure score** | 0.921 (0.909,0.934) | <0.001 | 0.846 (0.83, 0.862) | <0.001 |
| **Sleep health score** | 0.965 (0.947,0.983) | <0.001 | 0.923 (0.897, 0.950) | <0.001 |
| **Body mass index score** | 0.975 (0.961,0.989) | <0.001 | 0.989 (0.966, 1.013) | 0.365 |
| **Blood lipids score** | 0.975 (0.960,0.991) | 0.002 | 0.977 (0.952, 1.002) | 0.075 |
| **Blood glucose score** | 0.878 (0.861,0.894) | <0.001 | 0.855 (0.831, 0.879) | <0.001 |
| **Blood pressure score** | 0.902 (0.888,0.916) | <0.001 | 0.873 (0.852, 0.894) | <0.001 |
| ^a^Per 10-score increase. | | | | |
| ^b^Adjusted for age, gender, race/ethnicity, PIR, marital status, education level, alcohol consumption, health insurance and dental floss/device. | | | | |
| ^c^Reference category was no periodontitis. | | | | |
| Abbreviations: LC9, Life's Crucial 9; OR, odds ratio; CI, confidence interval; PHQ-9, Patient Health Questionnaire-9; HEI, Healthy Eating Index; PIR, poverty-to-income ratio | | | | |

**Table S6.** Linear regression analyses of the associations of Life's Crucial 9 scores with mean probing depth and mean clinical attachment loss.

| **Variable^a^** | **Mean probing depth^b^** | | **Mean clinical attachment lossb** | |
| --- | --- | --- | --- | --- |
|  | **β (95% CI)** | **P-value** | **β (95% CI)** | **P-value** |
| **LC9** | -0.097 (-0.108, -0.086) | <0.001 | -0.181 (-0.207, -0.155) | <0.001 |
| Q1 | Ref. | | Ref. | |
| Q2 | -0.066 (-0.107, -0.025) | 0.003 | -0.186 (-0.297, -0.075) | 0.002 |
| Q3 | -0.160 (-0.202, -0.117) | <0.001 | -0.374 (-0.471, -0.277) | <0.001 |
| Q4 | -0.320 (-0.362, -0.277) | <0.001 | -0.604 (-0.711, -0.496) | <0.001 |
| **P for trend** | <0.001 |  | <0.001 |  |
| **Health behaviours** | -0.016 (-0.018, -0.014) | <0.001 | -0.030 (-0.034, -0.026) | <0.001 |
| **Health factors** | -0.011 (-0.013, -0.009) | <0.001 | -0.021 (-0.025, -0.016) | <0.001 |
| **PHQ-9 score** | -0.006 (-0.017, 0.004) | 0.218 | -0.010 (-0.028, 0.008) | 0.288 |
| **HEI‐2015 diet score** | -0.023 (-0.028, -0.018) | <0.001 | -0.029 (-0.038, -0.020) | <0.001 |
| **Physical activity score** | -0.003 (-0.008, 0.001) | 0.112 | -0.010 (-0.016, -0.004) | 0.003 |
| **Nicotine exposure score** | -0.040 (-0.046, -0.034) | <0.001 | -0.082 (-0.095, -0.070) | <0.001 |
| **Sleep health score** | -0.021 (-0.027, -0.014) | <0.001 | -0.033 (-0.044, -0.021) | <0.001 |
| **Body mass index score** | -0.015 (-0.020, -0.010) | <0.001 | -0.004 (-0.013, 0.005) | 0.424 |
| **Blood lipids score** | -0.009 (-0.014, -0.003) | 0.005 | -0.010 (-0.023, 0.003) | 0.127 |
| **Blood glucose score** | -0.026 (-0.033, -0.020) | <0.001 | -0.067 (-0.081, -0.054) | <0.001 |
| **Blood pressure score** | -0.018 (-0.025, -0.012) | <0.001 | -0.059 (-0.070, -0.048) | <0.001 |
| ^a^Per 10-score increase. | | | | |
| ^b^Adjusted for age, gender, race/ethnicity, PIR, marital status, education level, alcohol consumption, health insurance and dental floss/device. | | | | |
| Abbreviations: LC9, Life's Crucial 9; OR, odds ratio; CI, confidence interval; PHQ-9, Patient Health Questionnaire-9; HEI, Healthy Eating Index; PIR, poverty-to-income ratio | | | | |

**Table S7.** Logistic regression analyses of the association Between Life's Crucial 9 score and periodontitis in samples without missing covariate data

| **Variable^a^** | **Unadjusted model** | | **Adjusted modelb** | |
| --- | --- | --- | --- | --- |
|  | **OR (95%CI)** | **P-value** | **OR (95%CI)** | **P-value** |
| **LC9** | 0.701 (0.666, 0.738) | <0.001 | 0.850 (0.800, 0.903) | <0.001 |
| Q1 | Ref. | | Ref. | |
| Q2 | 0.653 (0.561, 0.760) | <0.001 | 0.762 (0.645, 0.901) | 0.004 |
| Q3 | 0.495 (0.422, 0.579) | <0.001 | 0.680 (0.554, 0.835) | 0.001 |
| Q4 | 0.305 (0.245, 0.378) | <0.001 | 0.605 (0.483, 0.759) | <0.001 |
| **P for trend** | <0.001 |  | <0.001 |  |
| **Health behaviours** | 0.952 (0.944, 0.960) | <0.001 | 0.971 (0.962, 0.979) | <0.001 |
| **Health factors** | 0.953 (0.944, 0.962) | <0.001 | 0.985 (0.972, 0.997) | 0.021 |
| **PHQ9 score** | 0.971 (0.933, 1.010) | 0.149 | 1.035 (0.991, 1.081) | 0.133 |
| **HEI‐2015 diet score** | 0.963 (0.942, 0.984) | 0.001 | 0.986 (0.965, 1.007) | 0.199 |
| **Physical activity score** | 0.977 (0.963, 0.990) | 0.002 | 0.999 (0.984, 1.014) | 0.885 |
| **Nicotine exposure score** | 0.883 (0.868, 0.898) | <0.001 | 0.897 (0.881, 0.913) | <0.001 |
| **Sleep health score** | 0.935 (0.911, 0.960) | <0.001 | 0.975 (0.947, 1.004) | 0.106 |
| **Body mass index score** | 0.972 (0.955, 0.989) | 0.003 | 0.992 (0.972, 1.012) | 0.429 |
| **Blood lipids score** | 0.964 (0.945, 0.983) | 0.001 | 0.988 (0.962, 1.015) | 0.393 |
| **Blood glucose score** | 0.860 (0.840, 0.882) | <0.001 | 0.943 (0.916, 0.970) | 0.001 |
| **Blood pressure score** | 0.902 (0.882, 0.923) | <0.001 | 0.975 (0.950, 1.000) | 0.061 |
| ^a^Per 10-score increase | | | | |
| ^b^Adjusted for age, gender, race/ethnicity, PIR, marital status, education level, alcohol consumption, health insurance and dental floss/device. | | | | |
| Abbreviations: LC9, Life's Crucial 9; OR, odds ratio; CI, confidence interval; PHQ-9, Patient Health Questionnaire-9; HEI, Healthy Eating Index; PIR, poverty-to-income ratio | | | | |

| Variable^a^ | Unadjusted model | | Adjusted modelb | |
| --- | --- | --- | --- | --- |
|  | OR (95%CI) | P-value | OR (95%CI) | P-value |
| **LC9** | 0.694 (0.657, 0.732) | <0.001 | 0.840 (0.786, 0.898) | <0.001 |
| Q1 | Ref. | | Ref. | |
| Q2 | 0.706 (0.589, 0.846) | 0.001 | 0.829 (0.677, 1.015) | 0.082 |
| Q3 | 0.511 (0.429, 0.607) | <0.001 | 0.695 (0.556, 0.869) | 0.004 |
| Q4 | 0.306 (0.244, 0.383) | <0.001 | 0.615 (0.483, 0.784) | 0.001 |
| **P for trend** | <0.001 |  | <0.001 |  |
| **Health behaviours** | 0.948 (0.940, 0.956) | <0.001 | 0.966 (0.957, 0.975) | <0.001 |
| **Health factors** | 0.954 (0.944, 0.963) | <0.001 | 0.986 (0.973, 0.999) | 0.048 |
| **PHQ9 score** | 0.962 (0.922, 1.003) | 0.074 | 1.034 (0.986, 1.084) | 0.178 |
| **HEI‐2015 diet score** | 0.958 (0.938, 0.979) | <0.001 | 0.981 (0.960, 1.002) | 0.086 |
| **Physical activity score** | 0.970 (0.957, 0.983) | <0.001 | 0.990 (0.976, 1.004) | 0.160 |
| **Nicotine exposure score** | 0.883 (0.867, 0.900) | <0.001 | 0.897 (0.879, 0.916) | <0.001 |
| **Sleep health score** | 0.925 (0.901, 0.949) | <0.001 | 0.968 (0.940, 0.997) | 0.042 |
| **Body mass index score** | 0.966 (0.949, 0.984) | 0.001 | 0.992 (0.971, 1.014) | 0.495 |
| **Blood lipids score** | 0.970 (0.949, 0.992) | 0.009 | 0.993 (0.966, 1.022) | 0.655 |
| **Blood glucose score** | 0.855 (0.835, 0.877) | <0.001 | 0.938 (0.910, 0.966) | <0.001 |
| **Blood pressure score** | 0.906 (0.885, 0.928) | <0.001 | 0.979 (0.952, 1.007) | 0.145 |
| ^a^Per 10-score increase | | | | |
| ^b^Adjusted for age, gender, race/ethnicity, PIR, marital status, education level, alcohol consumption, health insurance and dental floss/device. | | | | |
| Abbreviations: LC9, Life's Crucial 9; OR, odds ratio; CI, confidence interval; PHQ-9, Patient Health Questionnaire-9; HEI, Healthy Eating Index; PIR, poverty-to-income ratio | | | | |

**Table S8.** Logistic regression analyses of the associations between Life's Crucial 9 score and periodontitis using dietary recall subsample weight

**Table S9.** Logistic regression analyses of the association between mediating variables and periodontitis

| **Outcome: Periodontitis** | **OR (95%CI)^a^** | **P-value** |
| --- | --- | --- |
| **ABSI (per 0.01 units increase)** | 1.506 (1.283, 1.768) | <0.001 |
| **WWI** | 1.256 (1.147, 1.375) | <0.001 |
| **SII (per 100 units increase)** | 1.045 (1.021, 1.070) | 0.001 |
| **SIRI** | 1.181 (1.096, 1.273) | <0.001 |
| ^a^Adjusted for age, gender, race/ethnicity, PIR, marital status, education level, alcohol consumption, health insurance and dental floss/device. | | |
| Abbreviations: OR, odds ratio; CI, confidence interval; LC9, Life's Crucial 9; ABSI, a body shape index; WWI, weight-adjusted-waist index; SII, Systemic Immune-Inflammation Index; SIRI, System Inflammation Response Index; | | |

**Table S10.** Linear regression analyses of the association between LC9 score and mediating variables

| **Outcome: Mediating variables** | **β (95%CI)^a^** | **P-value** |
| --- | --- | --- |
| **LC9^b^ - ABSI *100** | -0.048 (-0.057, -0.038) | <0.001 |
| **LC9^b^ - WWI** | -0.228 (-0.245, -0.211) | <0.001 |
| **LC9^b^ -S II/100** | -0.293 (-0.354, -0.231) | <0.001 |
| **LC9^b^ - SIRI** | -0.069 (-0.084, -0.053) | <0.001 |
| ^a^Adjusted for age, gender, race/ethnicity, PIR, marital status, education level, alcohol consumption, health insurance and dental floss/device. | | |
| ^b^Per 10-score increase. | | |
| Abbreviations: LC9, Life's Crucial 9; CI, confidence interval; LC9, Life's Crucial 9; ABSI, a body shape index; WWI, weight-adjusted-waist index; SII, Systemic Immune-Inflammation Index; SIRI, System Inflammation Response Index; | | |
